# Impaired Cellular Immunity to SARS-CoV-2 in Severe COVID-19 Patients

**DOI:** 10.3389/fimmu.2021.603563

**Published:** 2021-02-02

**Authors:** Ling Ni, Meng-Li Cheng, Yu Feng, Hui Zhao, Jingyuan Liu, Fang Ye, Qing Ye, Gengzhen Zhu, Xiaoli Li, Pengzhi Wang, Jing Shao, Yong-Qiang Deng, Peng Wei, Fang Chen, Cheng-Feng Qin, Guoqing Wang, Fan Li, Hui Zeng, Chen Dong

**Affiliations:** ^1^Institute for Immunology and School of Medicine, Tsinghua University, Beijing, China; ^2^Center for Human Disease Immuno-monitoring, Beijing Friendship Hospital, Beijing, China; ^3^College of Basic Medical Science, Jilin University, Changchun, China; ^4^Department of Virology, State Key Laboratory of Pathogen and Biosecurity, Institute of Microbiology and Epidemiology, Academy of Military Medical Sciences, Beijing, China; ^5^Beijing Ditan Hospital, Capital Medical University, and Beijing Key Laboratory of Emerging Infectious Diseases, Beijing, China; ^6^Department of Hematology, Chui Yang Liu Hospital Affiliated to Tsinghua University, Beijing, China; ^7^Department of Cardiology, Chui Yang Liu Hospital Affiliated to Tsinghua University, Beijing, China; ^8^China-Japan Union Hospital, Jilin University, Changchun, China; ^9^Beijing Key Lab for Immunological Research on Chronic Diseases, Beijing, China

**Keywords:** interferon gamma, T cells, neutralization antibody, adaptive immunity, acute respiratory distress syndrome, SARS-CoV-2

## Abstract

The high infection rate and rapid spread of severe acute respiratory syndrome coronavirus 2 (SARS-CoV-2) make it a world-wide pandemic. Individuals infected by the virus exhibited different degrees of symptoms, and most convalescent individuals have been shown to develop both cellular and humoral immune responses. However, virus-specific adaptive immune responses in severe patients during acute phase have not been thoroughly studied. Here, we found that in a group of COVID-19 patients with acute respiratory distress syndrome (ARDS) during hospitalization, most of them mounted SARS-CoV-2-specific antibody responses, including neutralizing antibodies. However, compared to healthy controls, the percentages and absolute numbers of both NK cells and CD8^+^ T cells were significantly reduced, with decreased IFNγ expression in CD4^+^ T cells in peripheral blood from severe patients. Most notably, their peripheral blood lymphocytes failed in producing IFNγ against viral proteins. Thus, severe COVID-19 patients at acute infection stage developed SARS-CoV-2-specific antibody responses but were impaired in cellular immunity, which emphasizes on the role of cellular immunity in COVID-19.

## Introduction

Identified in December, 2019, coronavirus disease 2019 (COVID-19) caused by severe acute respiratory syndrome coronavirus 2 (SARS-CoV-2) (1) has become a global public health threat. As of July 17th, 2020, 13,378,853 global cases were confirmed, of which 580,045 patients died (https://www.who.int/emergencies/diseases/novel-coronavirus-2019/situation-reports). The high infection rate and rapid spread make it a world-wide emergency (2). However, up to date, there is no specific anti-viral medicine or vaccine available to prevent or treat COVID-19 patients and the current standard care relies on supportive treatments.

Approximately 80% of COVID-19 cases are asymptomatic or manifest mild symptoms resembling simple upper respiration tract infection. The remaining COVID-19 patients show severe or critical symptoms with severe pneumonia and acute respiratory distress syndrome (ARDS) (3). How the immune system modifies and controls the infection is still not well understood, which may underscore the different degrees of symptoms among the population. Several papers were reported on the adaptive immune responses in the recovered mild COVID-19 patients. We detected SARS-CoV-2-specific antibodies and T cells in convalescent individuals (4). Wu et al. found that around 94% of COVID-19 convalescent individuals had neutralizing antibodies (5). Grofoni et al. also found that ~70% and 100% of COVID-19 convalescent subjects with mild and severe symptoms developed SARS-CoV-2-specific CD8^+^ and CD4^+^ T cells, respectively (6).

Currently, there is very limited understanding on immune responses in severe COVID-19 patients during hospitalization. In a retrospective study, Chen et al. observed that absolute numbers of T lymphocytes, both CD4^+^ and CD8^+^, were markedly lower in severe patients than those in moderate cases (7). In contrast to the aforementioned study, Zheng et al. did not observe reduced lymphocytes in the severe disease group, but T cells showed elevated exhaustion levels and reduced functional heterogeneity (8). Using megapools of overlapping or prediction-based peptides covering the SARS-CoV-2 proteome, Weiskopf et al. found CD4^+^ and CD8^+^ T cells showed activation marker expression in 10 out of 10 and eight out of 10 patients with severe COVID-19 during hospitalization, respectively (9), though their function was not characterized. For humoral responses, Lynch et al. found that antibody responses were higher in patients with severe than mild disease (10). And high levels of neutralizing antibodies were induced 10 days post onset in both mild and severe patient, which were higher in severe group (11). Thus, virus-specific adaptive immune responses to COVID-19 in severe patients are not well understood.

In order to understand immune responses and the mechanism underlying the pathogenesis of severe COVID-19, we collected blood samples from 10 patients with ARDS, analyzed their cellular and humoral responses specific to SARS-CoV-2, and compared severe and convalescent patients. Our data reveal defective cellular immunity associated with severe COVID-19. This study provides new insights on the precise treatment of COVID-19 patients and evaluation of candidate vaccines.

## Materials and Methods

### COVID-19 Patient Blood Samples

The blood samples of severe COVID-19 patients defined as severe lower respiratory tract infection or pneumonia with fever plus any one of the following: tachypnea, respiratory distress, or oxygen saturation less than 93% on room air according to the guidelines released by the national Health Commission of China were obtained from Ditan hospital in Beijing. The convalescent patient results were reported in our previous study (4), and used to compare with the data of the severe patients. All procedures were in accordance with the ethical standards of the responsible committee on human experimentation (the institutional review board at Tsinghua University and at Ditan hospital) and with the Helsinki Declaration of 1975, as revised in 2000. All studies were approved by the Medical Ethical Committee at Tsinghua University. Informed consent was obtained from all subjects for being included in the study. All patient data were anonymized before study inclusion.

### Cell Lines

Angiotensin converting enzyme 2 (ACE2)-positive Huh-7 cells originally taken from a liver tumor in a Japanese male were cultured in DMEM supplemented with 10% FBS. Cells were grown at 37°C in a 5% CO2 setting.

### Expression and Purification of Recombinant Proteins

The recombinant His-tagged nucleocapsid protein (NP) of SARS-CoV-2 was expressed in E. coli by a T7 expression system, with 1 mM IPTG induction at 37°C for 4 h. The recombinant His-tagged receptor-binding domain (RBD) of spike protein (S-RBD) (amino acids 319-541) was expressed by a mammalian system in 293F cells. Purified proteins were identified by SDS-PAGE gels and stained with Coomassie blue. The purities of the His-tagged NP and His-tagged S-RBD were around 90% and the endotoxin levels of NP and S-RBD were 0.01 and 0.02 EU per µg, respectively.

### Isolation of Peripheral Blood Mononuclear Cells

PBMCs were isolated from anti-coagulant blood using Ficoll-Hypaque gradients (GE Healthcare Life Sciences, Philadelphia, PA) as previously described (12) under the biosafety level 3 facility in AMMS. The whole blood was centrifuged for 5 min to separate plasma and cells. Then blood cells diluted with PBS were gently layered over an equal volume of Ficoll in a tube and centrifuged for 30–40 min at 400–500 g without brake. Four layers formed with the second containing PBMCs. A Pasteur pipette was used to obtain PBMCs in the second layer. The PBMCs were then cryopreserved for future study.

### Anti-SARS-CoV-2 IgG/IgM ELISA

For SARS-CoV-2-specific IgM/IgG testing, 96-well ELISA plates were coated overnight with recombinant NP and S-RBD (80 ng/well) at 4°C. Plates were washed and the sera from COVID-19 patients were incubated for 1 h at 37°C. After washing, an anti-Human IgG-biotin conjugated monoclonal antibody (Cat. SSA009, Sino Biological Inc., Wayne, PA) and streptavidin-HRP were used at a dilution of 1: 5000 and 1:250, respectively, and anti-human IgM-HRP conjugated monoclonal antibody (Cat. bs-0345G-HRP, Biosynthesis Biotechnology Inc. Beijing, China) was used at a dilution of 1:1000. TMB substrate solution was added, followed by extensive wash. The OD value at 450 nm was calculated. The area under the curve (AUC) was calculated by Prism 8 (GraphPad). As a second analytical approach (6), the serum from one convalescent mild COVID-19 patient was used as a positive control standard. In order to quantify the amount of anti-NP/S-RBD IgG or anti-NP/S-RBD IgM present in each specimen, the positive control standard was run on each plate to calculate antibody titers (relative units) for all samples using non-linear regression interpolations.

### Anti-SARS-CoV-2 IgG1/IgG3 ELISA

For IgG1/IgG2/IgG3 test, 96 well ELISA plates were coated (80 ng/well) overnight with recombinant NP and S-RBD at 4°C. Plates were washed and the sera from COVID-19 patients were incubated for 1 h at 37°C. After washing, anti-Human IgG1-HRP conjugated monoclonal antibody (Cat. C030248, BaiaoTong Experiment Center, LY), anti-Human IgG2-HRP conjugated monoclonal antibody (Cat. C030245, BaiaoTong Experiment Center, LY) and anti-human IgG3-HRP conjugated monoclonal antibody (Cat.C030246, BaiaoTong Experiment Center, LY), all validated by the company for their specificity, were used at a dilution of 1:4000 for 1 h at RT. TMB substrate solution was added, followed by washing. The OD value at 450 nm was calculated. The area under the curve (AUC) was calculated by Prism 8 (GraphPad).

### Neutralizing Antibody Assay

Pseudovirus expressing the SARS-CoV-2 S protein was produced. pNL43Luci and GP-pCAGGS were co-transfected into 293T cells. 48 h later, SARS-CoV-2 pseudovirus-containing supernatants were mixed with at least 6 serially diluted serum samples from the COVID-19 patients at 37°C for 1 h. Then the mixtures were transferred to 96-well plates containing monolayers of Huh-7 cells (13). Three hours later, the medium was replaced. After incubation for 48 h, the cells were washed, harvested in lysis buffer and analyzed for luciferase activity by the addition of luciferase substrate. Inhibition rate = [1-(the sample group- the cell control group)/(the virus control group- the cell control group)] x 100%. The neutralizing antibody titer (NAT50) were calculated by performing S-fit analysis *via* GraphPad Prism 7 software.

### Interferon Gamma ELISpot

IFN-γ-secreting T cells were detected by Human IFNγ ELISpot^pro^ kits (MABTECH AB, Sweden) as indicated in the manufacture protocol. The cryopreserved PBMCs after thaw were plated in duplicate at 150k/well and then incubated 48 h with 1 µM of recombinant proteins. Anti-CD3 antibody (0.1 µg/ml) was used as a positive control, while medium alone as negative controls. Spots were then counted using an AID ELISpot Reader System (iSpot, AID GmbH). The number of spots was converted into the number of spots per million cells. Mean spots of the negative wells were subtracted from the experimental wells as well as positive wells.

### Cell Surface Staining

PBMCs were washed with PBS plus 2% FBS (Gibco, Grand Island, NY), and then Fc blocking reagent (Miltenyi Biotec, Inc., Auburn, CA) was added, followed by extensive wash. Cells were then incubated for 30 min on ice with anti-CD3 (OKT3) (BioLegend), anti-CD8 (SK1) (BD), anti-CD56 (HCD56) (BioLegend) and live/dead fixable aqua dye (eF660, eBioscience), washed twice with PBS plus 2% FBS and then stored at 4°C until acquired by FACS Verse (BD Biosciences, San Jose, CA). Data were analyzed using FlowJo software (Version 10.0.8, Tree Star Inc., Ashland, Or).

### Intracellular Cytokine Staining

The PBMCs were stimulated with phorbol myristate acetate (PMA)/Ionomycin for 4 h with GolgiPlug (brefeldin A, BD). For the flow cytometry (FACS) staining, dead cells were first stained with live/dead fixable aqua dye. Next, surface markers were stained. Cells were then washed, fixed with Cytofix/CytopermTM (BD Biosciences) and stained with PE-Cy7-anti-IFNγ, PE-anti-tumor necrosis factor alpha (TNFα) and FITC-anti-IL-17A. The samples were acquired on FACS Verse (BD Biosciences, San Jose, CA) and analyzed with FlowJoTM v.10 software for Mac (Version 10.0.8, Tree Star Inc., Ashland, Or).

### Statistical Analysis

Prism 8 software is used for statistical analysis. Student’s t test was performed for two-group analysis. Pearson’s correlation coefficients were calculated. *P* values less than 0.05 were considered to be statistically significant.

## Results

### Analysis of Functional SARS-CoV-2-Specific Antibodies in Severe COVID-19 Subjects

To understand the immune responses to SARS-CoV-2 in severe patients, we studied 10 patients with ARDS. Their clinical and pathological characteristics are shown in **Table S1**. All the patients were hospitalized at Beijing Ditan Hospital and showed severe symptoms *via* CT scan and were positive in SARS-CoV-2 nucleic acid testing. The mean age was 57.5 years and half of them were female. Among them, eight (80%) showed lymphopenia. As of today, one patient (Pt#9) passed away and the remaining ones had recovered and were discharged from hospital. The blood samples were obtained within 20 days post symptom onset and the detailed sampling day for each patient was also shown in **Table S1**. Human AB serum collected from healthy male AB donors in the US (GemCell, CA) was used as a negative control. Additionally, sera from nine healthy donors were obtained before the SARS-CoV-2 outbreak (HD#1-9). Five additional healthy donors (HD#10-14) without SARS-CoV-2 infection were analyzed in our neutralizing and T cell assays.

**Figure 1:**
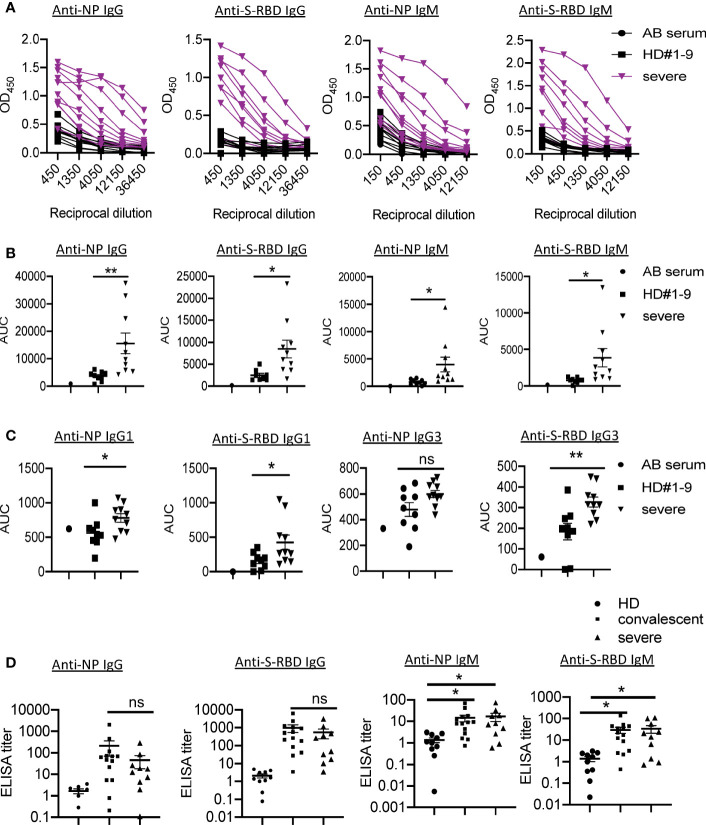
Presence of SARS-CoV-2 NP- and S-RBD-specific antibodies in severe COVID-19 patients. **(A)** Titration of individual serum samples. **(B)** Data from the same experiments as **(A)** were presented as area under curve (AUC). **(C)** IgG isotypes of 10 COVID-19 patients to recombinant NP and S-RBD. **(D)** Serum ELISA titers to NP and S-RBD in sera from convalescent individuals (n=14), severe patients (n=10) and HD (n=10). The experiment was performed in duplicates. NP, nucleocapsid protein. S-RBD, receptor binding domain of spike protein. HD, healthy donor; Pt, patient; AUC, area under curve. HD#1-9, the sera were collected before SARS-CoV-2 outbreak. *P<0.05, 0.05<**P<0.001. ns, not significant.

Using sera from patients and healthy donors, IgG and IgM specific to SARS-CoV-2 NP and S-RBD antigens were analyzed using ELISA assay previously reported (4). The individual serum samples were performed by serial dilutions to calculate the AUC values (**Figure 1A**). Compared with healthy donors, patients with severe COVID-19 showed significantly elevated anti-NP IgG AUC values (**Figure 1B**). The AUC values of anti-S-RBD IgG in severe cases were also significantly increased compared to those in healthy controls. Serum NP- and S-RBD-specific IgM antibodies showed significantly higher AUC values in severe COVID-19 patients than in healthy controls (**Figure 1B**). Notably, patients #1, 4, and 7 did not develop NP- and S-RBD -specific antibody responses, including IgM and IgG. As shown in **Figure 1C**, anti-NP and S-RBD IgG in severe patients was mainly IgG1 isotype, as in convalescent individuals (4). We did not detect IgG2 to either NP or S-RBD proteins (data not shown).

In order to understand the pathogenic mechanisms in severe patients, we compared the levels of virus-specific IgG or IgM in these patients with those in convalescent individuals. Serum from one convalescent COVID-19 patient with high levels of SARS-CoV-2 IgG/IgM AUC values was used as a positive control standard to calculate the antibody titers (relative units) for all samples using non-linear regression interpolations (6). Of note, we observed no significant differences in anti-NP/S-RBD IgG or IgM between these two groups (**Figure 1D**).

**Figure 2:**
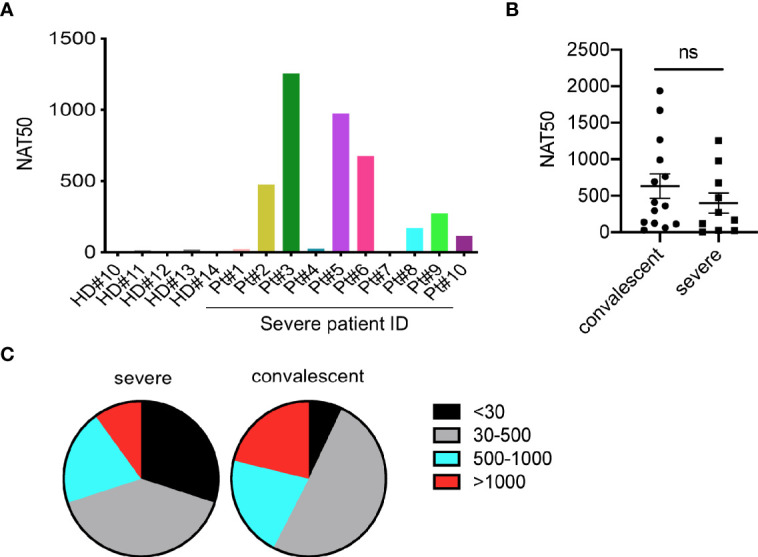
Measurement of neutralizing antibodies in severe patients. **(A)** Neutralizing antibody titers of 10 severe COVID-19 patients. **(B)** Comparison of NAT50 between convalescent subjects (n=14) and severe patients (n=10). Date are presented as Mean ± SEM. **(C)** Pie plot showing NAT50 range in severe and convalescent patients. HD, healthy donor; Pt, patient; NAT50, neutralizing antibody titers. ns, not significant.

To determine the neutralization capacity in sera from patients with severe COVID-19, we performed pseudovirus particle-based neutralization assay as previously described (4). As shown in **Figure S1** and **Figure 2A**, patients #1, 4, and 7, did not produce neutralizing antibodies, while patient #3 had a high NAT50 (NAT50>1000). Most severe patients (70%) had protective humoral immunity to SARS-CoV-2. Notably, sera from severe patients did not show significantly reduced NAT50 values than convalescent sera (**Figure 2B**). About 30% of severe patients and 8% of convalescent individuals did not produce neutralizing antibodies, respectively (**Figure 2C**). Around 40%, 20% and 10% of severe patients showed low (NAT50: 30-500), medium (NAT50: 500-1000) and high (NAT50: >1000) NAT50, respectively, while 50%, 21%, and 21% in the convalescent group did.

Taken together, these findings indicate that most severe COVID-19 patients mounted IgG and IgM responses specific to SARS-CoV-2 proteins, NP and S-RBD. Moreover, these patients had serum neutralizing antibodies to SARS-CoV-2 infection. However, the levels of humoral immune responses varied among the patients.

### Decreased Lymphocyte Numbers and Function in Severe COVID-19 Subjects

**Figure 3:**
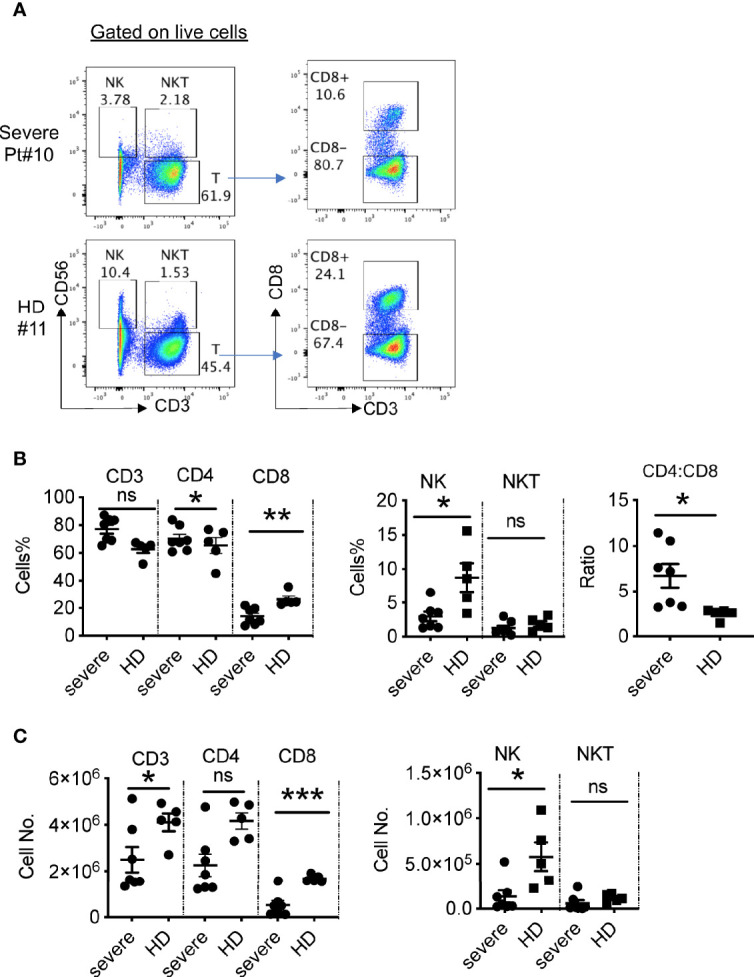
Phenotypic analysis of blood lymphocytes in severe COVID-19 patients. **(A)** Phenotypic analysis of PBMCs from one representative COVID-19 patient. **(B)** Summarized data on the frequencies of different immune cell subsets in COVID-19 patients and the ratio of CD4:CD8 T cells. **(C)** Summarized data on the absolute numbers of different immune cell subsets in COVID-19 patients. HD, healthy donors (HD#10-14); Pt, patients (n=7). Data are presented as Mean ± SEM. *P<0.05, 0.05<**P<0.001, ***P<0.001. ns, not significant.

To analyze cellular immune responses to SARS-CoV-2, PBMCs from 10 patients with severe COVID-19 and five healthy donors were phenotypically analyzed by flow cytometry (**Figure 3A**). We did not detect live PBMCs in three out of 10 patients (Pts# 3, 4, and 5), possibly due to technical issues during cryopreservation. The gating strategy is shown in the **Figure S2**. Compared to healthy donors, there was a significant decrease in the percentages of NK cells in the severe patients, but similar frequencies of NKT cells (**Figure 3B**). Although there was a trend towards increased frequencies of T cells (CD3^+^CD56^-^) in the patient blood, the absolute numbers of T cells were significantly decreased compared to those in blood from healthy controls (**Figure 3C**). Notably, the percentages and absolute numbers of CD8^+^ T cells were reduced dramatically, while the frequency of CD4^+^ T cells was elevated compared with those in healthy donors. As a result, the CD4:CD8 ratios were significantly higher in severe patients than in the healthy donors (6.7 ± 1.3 in COVID-19 vs. 2.5 ± 0.3 in HD, P=0.0226) (**Figure 3B** and **Table S2**). Thus, the severe patients exhibited reduced cytotoxic lymphocytes, both NK and CD8^+^ T cells.

**Figure 4:**
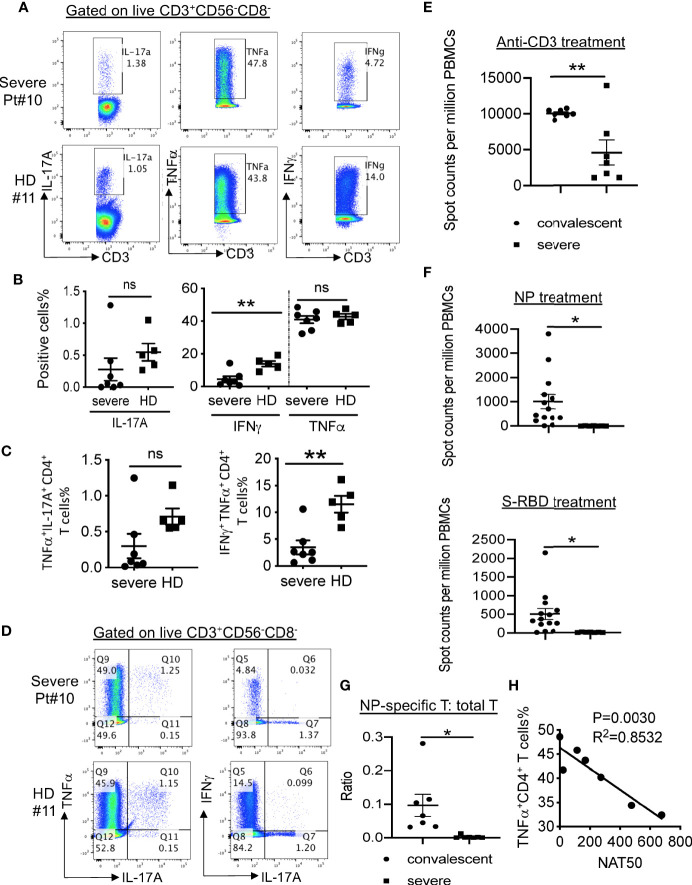
SARS-CoV-2-specific T cell responses in COVID-19 patients. **(A)** Intracellular cytokine staining of CD3^+^CD56^-^CD8^-^ T cells. **(B)** Summarized data on the frequencies of cytokine-producing CD3^+^CD56^-^CD8^-^ T cells as indicated. **(C)** Summarized data on the frequencies of polyfunctional CD3^+^CD56^-^CD8^-^ T cells as indicated. **(D)** Representative FACS plots showing polyfunctional CD3^+^CD56^-^CD8^-^ T cells. **(E)** IFNγ ELISpot analysis of PBMCs from severe COVID-19 patients (n=7) and convalescent individuals (n=7) to anti-CD3 antibody. **(F)** IFNγ ELISpot analysis of PBMCs from severe COVID-19 patients (n=7) and convalescent individuals (n=14) to and viral proteins. The experiment with patients was performed in duplicates. **(G)** The ratios of IFNγ-producing T cells in response to NP or anti-CD3 stimulation in both convalescent (n=14) and severe (n=7) patients. **(H)** Correlation between NAT50 and the percentage of TNFα^+^ CD4^+^ T cells. Date are presented as Mean ± SEM. *P<0.05, 0.05<**P<0.001. ns, not significant.

To further assess the function of the T cells in the severe patients, we stimulated T cells with PMA and ionomycin and then measured cytokine production. As shown in **Figure 4A, B**, CD4^+^ T cells from severe patients expressed significantly lower levels of IFNγ than those from healthy donors. We observed no apparent difference in the frequencies of TNFα-expressing CD4^+^ T cells, but significantly reduced percentages of IFNγ^+^ TNFα^+^ T cells (**Figures 4C, D**), consistent with a published report (7). Only two out of seven patients had detectable IL-17A^+^ CD4^+^ T cells, which expressed TNFα, but not IFNγ (**Figure 4D**).

Despite the decreased frequencies of CD8^+^ T cells in PBMCs from the patients, these CD8^+^ T cells expressed similar levels of cytokines IFNγ and TNFα as the ones from healthy donors (**Figures S3A, B**). In addition, similar frequencies of IFNγ^+^TNFα^+^ CD8^+^ T cells were found in severe patients and healthy donors (**Figure S3C**).

We also evaluated capacities of cytokine production in CD3^-^ cells, likely NK cells. As shown in **Figure S3D**, these cells in severe patients expressed significantly reduced levels of IFNγ and TNFα than those in healthy donors. We did not observe significant difference in terms of the frequency of IFNγ^+^ TNFα^+^ cells between these two groups (**Figure S3D**).

In summary, these findings indicate that the severe patients displayed a decrease in the numbers as well as function of peripheral lymphocytes, especially CD8^+^ T cells and NK cells.

### Lack of Cellular Immune Responses to SARS-CoV-2 in Severe COVID-19 Subjects

To further measure virus-specific cellular immunity, we treated PBMCs with recombinant NP and S-RBD, followed by IFNγ ELISpot analysis. As shown in **Figure 4E**, the absolute numbers of T cells produced IFNγ in response to anti-CD3 were decreased dramatically in PBMCs from severe patients than those from healthy donors. More strikingly, we did not detect any SARS-CoV-2-specific T cells in all the tested severe patients (**Figure 4F**), whereas one out of ten T cells on average could secret IFNγ after exposure to NP protein in convalescent individuals (**Figure 4G**).

Lastly, to explore how the antibody response related to cellular immunity in the severe patients, we performed correlative analysis. No significant correlations between NAT50 and the frequencies of cytokine-producing T cells (IFNγ^+^ CD4, IFNγ^+^ CD8, IL-17^+^ CD4, IL-17^+^ CD8, TNFα^+^ CD8) were noted (data not shown). Surprisingly, we found that NAT50 values negatively associated with the frequencies of TNFα-expressing CD4^+^ T cells (**Figure 4H**), suggesting that pro-inflammatory function of T cells may be enhanced due to insufficient antibody responses.

Taken together, the severe patients during acute phase failed in mounting SARS-CoV-2-specific T cell responses, though with development of humoral immune responses.

## Discussion

In this study, we characterized humoral and cellular immunity in severe COVID-19 patients during hospitalization. Although most patients developed anti-SARS-CoV-2 IgG and IgM responses, humoral immune responses varied widely among the patients. The percentages and absolute numbers of both NK cells and CD8^+^ T cells were significantly reduced, accompanied with decreased Th1 cell response in peripheral blood from severe patients. Most notably, we failed in detecting SARS-CoV-2-specific IFNγ production in these patients.

NAT50 in the severe patients varied widely ranging from zero to thousands. Although we did not understand the precise reason, viral load, patient sex and age, lymphocyte counts as well as patient immune health might contribute to antibody responses to SARS-CoV-2 infection. In addition, the NAT50 during acute infection positively correlated with anti-NP IgG AUC, but not anti-RBD IgG AUC (data not shown), which was in contrast to our previous study that NAT50 in convalescent sera from mild patients correlates with anti-S-RBD IgG AUC. Another study by Wang et al. also showed that anti-SARS-CoV-2 NP IgG level exhibited moderate correlation with NAT50 (11). The discrepancy may attribute to disease severity and stage. Moreover, the sample size may also cause this inconsistency.

TNFα contributes to T cell-dependent B-cell responses. Memory CD4^+^ T cells activated by cytokines, such as TNFα, provide help with the production of IgM, IgG and IgA (14). Moreover, TNFα secreted by CD4^+^ T cells serve as a costimulatory signal for B cells (15). However, we found that the NAT50 correlated with the percentage of TNFα^+^CD4^+^ T cells in a negative fashion. The underlying mechanism needs future investigation.

Most severe patients had reduced Th1 cell responses, consistent with the published report (7). Another report showed that peripheral blood of a severe COVID-19 patient had a strikingly high number of CCR6^+^ Th17 cells (16). In addition, several papers proposed use of therapies directed at Th17 cells and IL-17A signaling in treating COVID-19 patients (17–19). However, we observed no significant Th17 cell responses in most of the severe patients (5/7) with only one patient showing elevated IL-17A expression. Several possibilities may account for the discrepancy. One is different markers used for defining Th17 cells. The above-mentioned paper used CCR6 on CD4^+^ T cells to define Th17 cells, whereas we used IL-17A expression in CD4^+^ T cells. Another is different patient cohort and disease severity. Sample timing during disease course is another possibility, since T cell responses are dynamic. Given not all the severe COVID-19 patients had increased IL-17A expression, one may not want to treat all patients with IL-17A inhibitors.

5 healthy donors (HD#10-14) with a mean age of 29.4 years were included in our T cell assays, which are not age-matched with severe patients with a mean age of 57 years. Compared to healthy controls, severe patients showed reduced percentages of CD8^+^ T cells and IFNγ-expressing CD4^+^ T cells. Previously, Carr et al. found that frequencies of Th1 and CD8^+^ T cells were enhanced with age (20). Thus, the differences we observed between healthy controls and severe patients were unlikely caused by age. Lymphopenia is common in severe COVID-19 patients. The underlying cause could be that the blood lymphocytes were recruited to the respiratory tract or adhered to inflamed respiratory vascular endothelium (21), which requires extensive investigation. In this study, we found that the lymphopenia in severe patients was mainly in CD8^+^, but not CD4^+^ T cell compartment. However, IFNγ response in CD4^+^ T cells was greatly reduced. In animal models, lymphopenia enhanced T cell activation and proliferation (22). Thus, the relationship between lymphopenia and Th1 reduction in COVID-19 patients warrants further confirmation.

Antigens-specific IFNγ expression was not detected in severe patients, in contrast to the report by Weikokf et al. (9). In addition, Betts and colleagues observed higher levels of granzyme B and perforin in CD8^+^ cells from severe patients than those from HD (23). Several factors were responsible for this discrepancy. Firstly, we used antigen-mediated IFNγ ELISpot analysis to detect antigen-specific T cells, while the other group used T cell receptor-dependent activation marker (AIM) assay in measuring the expression of CD137 and CD69. Thus, antigen-specific T cells detected by AIM assay might not be functional. Secondly, different patient cohort and sample timing may account for the different results. Lastly, although the underlying mechanisms by which severe patients during acute phase failed in developing virus-specific effector T cells remained largely unknown, according to the findings by Zhou et al, dampened function of dendritic cells (DCs) might contribute to delayed T cell immune responses in severe COVID-19 patients (24). In this study, we used recombinant SARS-CoV-2 proteins (NP and S-RBD), not overlapping peptide pools (which were used by Weikokf et al.), to stimulate T cells. Impaired DC response to SARS-CoV-2, especially reduced capacity of processing pathogens, might lead to dampened antigen-specific T cell response. Of note, Crotty and colleagues recently showed that severe patients in acute phase mounted relatively normal antibody responses, but impaired T cell responses (25), in line with our conclusion.

In summary, we detected anti-SARS-CoV-2 antibody responses in most severe cases, while impaired cellular responses were observed in all severe COVID-19 patients. Our results thus provide insight in the pathogenesis of severe COVID-19. They suggest that induction of cellular immunity is vital in controlling SARS-CoV-2 infection, which also has implications in development of an effective vaccine.

## Data Availability Statement

The raw data supporting the conclusions of this article will be made available by the authors, without undue reservation.

## Ethics Statement

The studies involving human participants were reviewed and approved by The institutional review board at Tsinghua University and at Ditan hospital. The patients/participants provided their written informed consent to participate in this study.

## Author Contributions

LN and CD designed the research and analyzed the data. FY and FC collected clinical specimens with mild COVID-19. JL and HZe collected clinical specimens with severe COVID-19. Y-QD, XL, and QY did most of the experiments at a P3 laboratory. M-LC, YF, HZh, QY, LX and PWe performed some experiments or prepared key reagents. LN and CD analyzed the results. LN and CD wrote the manuscript. All authors contributed to the article and approved the submitted version.

## Funding

This work was supported in part by grants from National Key Research and Development Program of China (2016YFC1303900 to LN), Tsinghua University Spring Breeze Fund (2020Z99CFG008 to LN), Natural Science Foundation of China (31991173, 31821003 and 31991170 to CD), Beijing Municipal Science and Technology (Z181100001318007, Z181100006318015 and Z171100000417005 to CD), Zhejiang University Foundation (2020XGZX014 to CD), Science and Technology Development Plan Project of Jilin Province (20200901007SF to FL), Science and Technology Plan of Beijing Chaoyang District (CYSF2061 to FC) and Beijing Hospital Authority (DFL20191801 to HZe).

## Conflict of Interest

The authors declare that the research was conducted in the absence of any commercial or financial relationships that could be construed as a potential conflict of interest.

## Abbreviations

COVID-19, coronavirus disease 2019; SARS-CoV-2, severe acute respiratory syndrome coronavirus 2; ARDS, severe pneumonia and acute respiratory distress syndrome; ACE, angiotensin converting enzyme 2; NP, nucleocapsid protein; RBD, receptor-binding domain; S-RBD, receptor-binding domain of spike protein; PBMCs, peripheral blood mononuclear cells; AUC, area under the curve; NAT50, neutralizing antibody titer; IFNγ, interferon gamma; TNFα, tumor necrosis factor alpha; ICS, intracellular cytokine staining; PMA, phorbol myristate acetate; FACS, flow cytometry; DCs, dendritic cells.
