# Supplementary figures and images for: Impaired Cellular Immunity to SARS-CoV-2 in Severe COVID-19 Patients
Source: Front Immunol. 2021 Feb 2;12:603563. doi: 10.3389/fimmu.2021.603563 (PMC7884325; doi:10.3389/fimmu.2021.603563)

## Slide 1
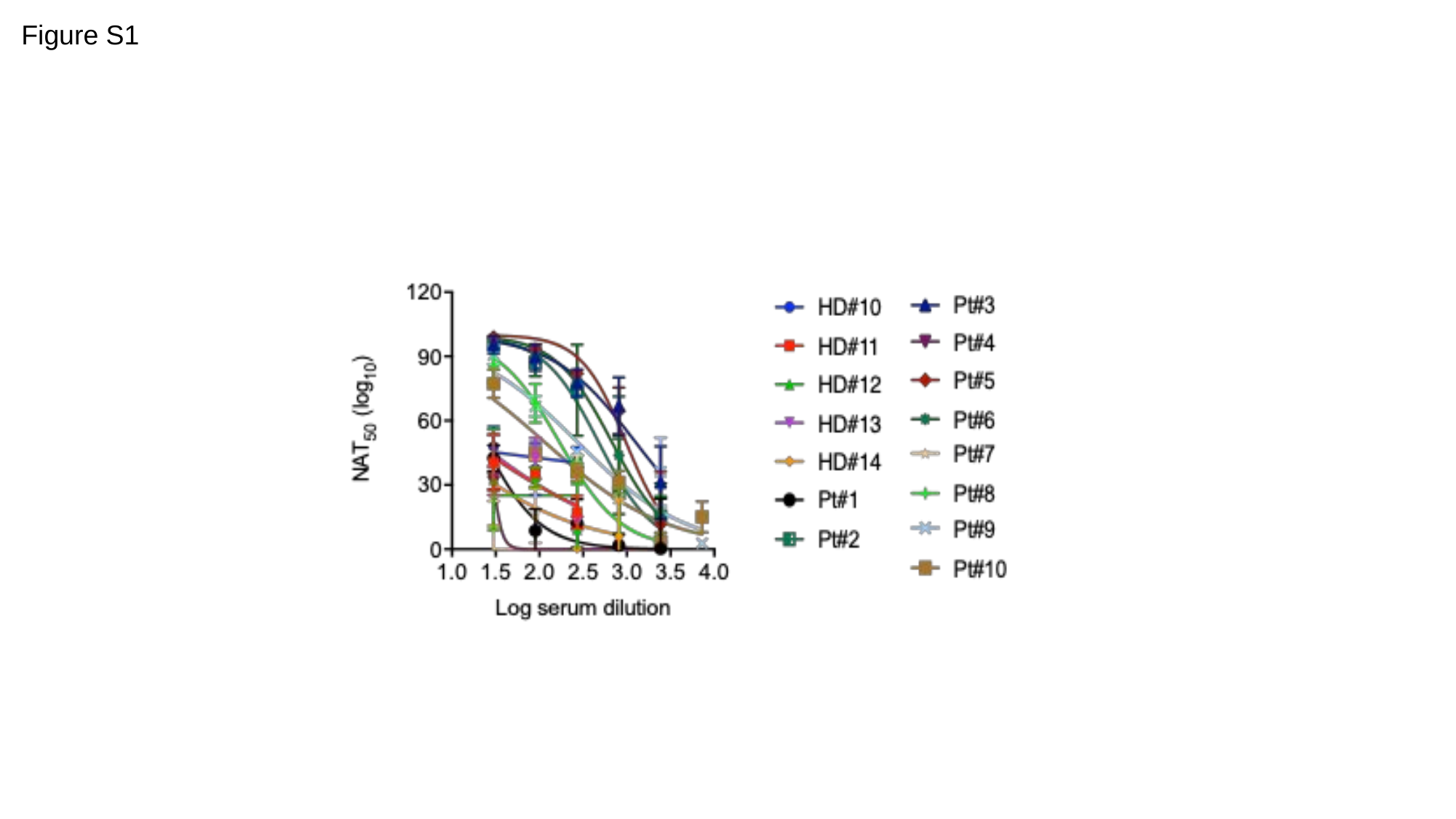

Figure S1

## Slide 2
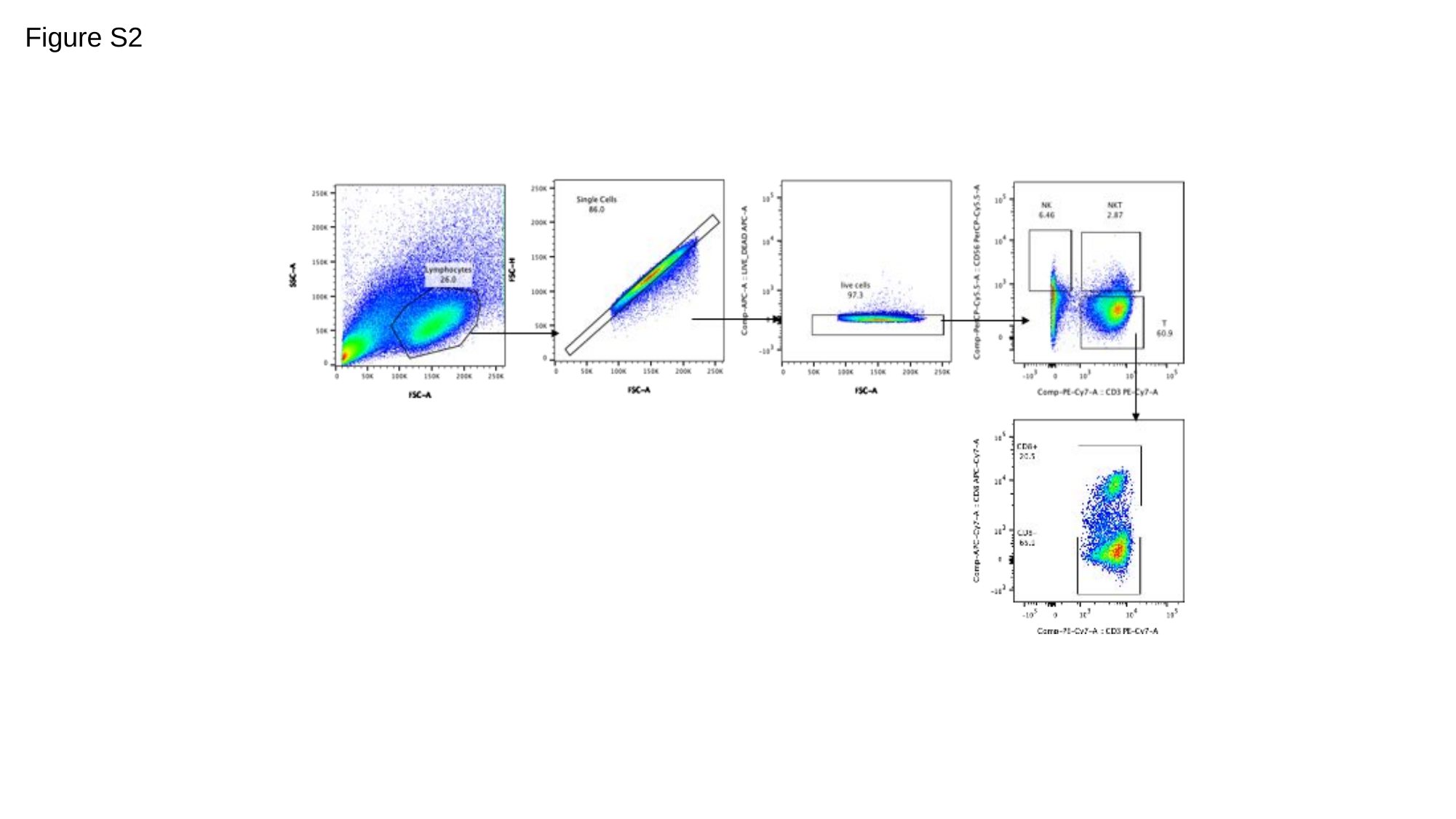

Figure S2

## Slide 3
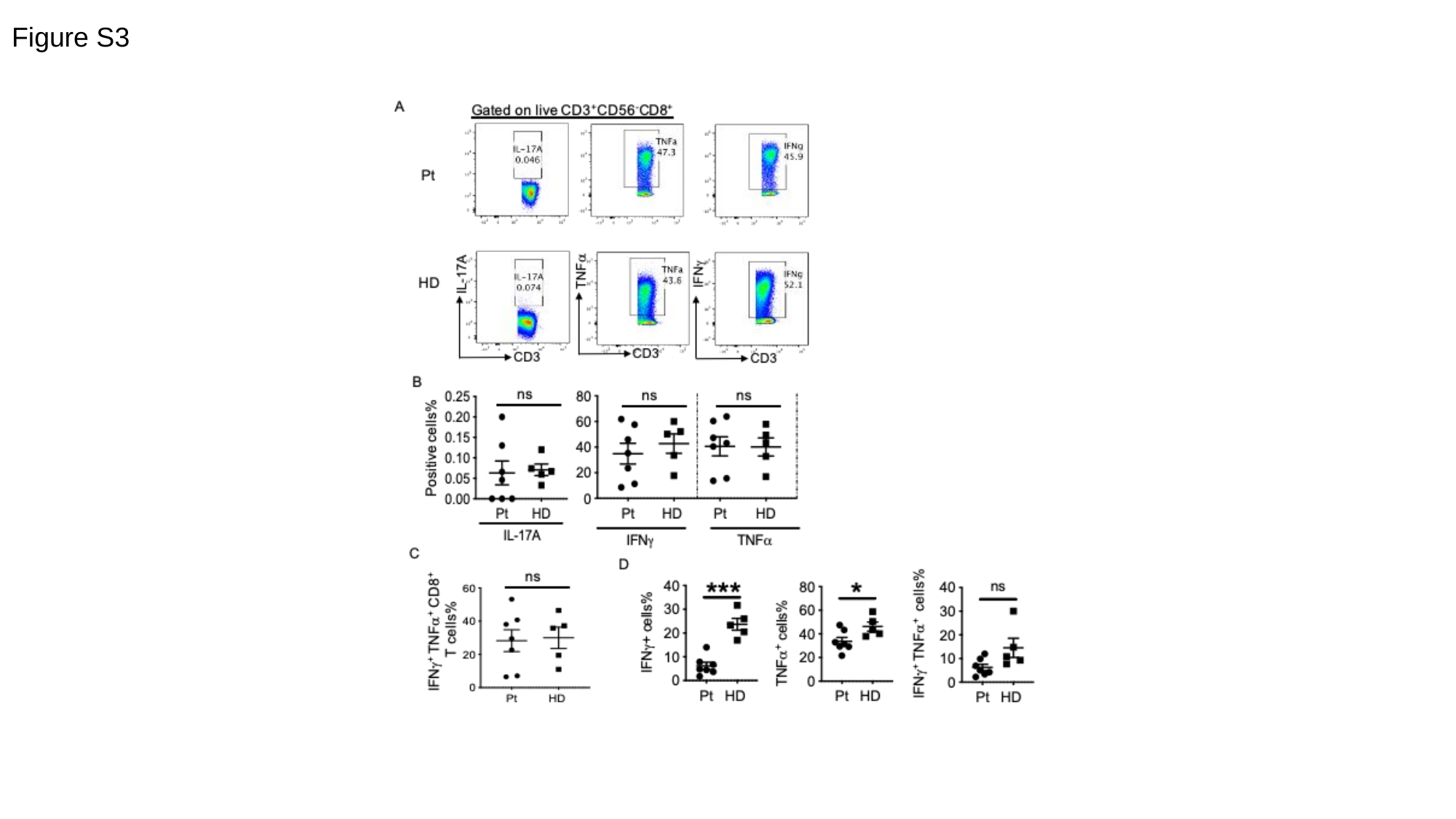

Figure S3

Supplement: Supplementary file 1 [file Presentation_1.pptx]
