# Supplementary material for: Impaired Cellular Immunity to SARS-CoV-2 in Severe COVID-19 Patients
Source: Front Immunol. 2021 Feb 2;12:603563. doi: 10.3389/fimmu.2021.603563 (PMC7884325; doi:10.3389/fimmu.2021.603563)
Supplement: Supplementary file 2 [file Table_1.docx]

Table S1

**Table S1 Clinical and pathological characteristics of the severe COVID-19 patients**

| **Pt#** | **Sex** | **Age** | **Fever** | **Fatigue** | **ARDS** | **Lymphocyte Count** | **Days in hospital** | **Sampling day post onset** | **BT NA test** | **Discharge CT scan** | **Discharge NA test** |
| --- | --- | --- | --- | --- | --- | --- | --- | --- | --- | --- | --- |
| #1 | F | 69 | yes | no | yes | 0.65X10^9^/L | 43 | 13 | P | improvement | N |
| #2 | M | 55 | yes | no | yes | 1.24X10^9^/L | 27 | 18 | P | improvement | N |
| #3 | M | 37 | yes | no | yes | 0.53X10^9^/L | 47 | 19 | P | improvement | N |
| #4 | M | 52 | yes | no | yes | 0.94X10^9^/L | 39 | 8 | P | improvement | N |
| #5 | F | 58 | yes | no | yes | 0.78X10^9^/L | 50 | 12 | P | improvement | N |
| #6 | M | 40 | yes | no | yes | 1.13X10^9^/L | 51 | 11 | P | improvement | N |
| #7 | F | 70 | yes | no | yes | 0.75X109/L | 36 | 11 | P | improvement | N |
| #8 | F | 66 | yes | Yes | yes | 0.69X10^9^/L | 43 | 2 | P | improvement | N |
| #9 | M | 66 | yes | No | yes | 0.92X10^9^/L | 82 | 4 | P | N/A | N/A |
| #10 | F | 62 | yes | yes | yes | 0.26X10^9^/L | 43 | 16 | P | improvement | N |

Notes: pt, patient; F, female; M, male; ARDS, acute respiratory distress syndrome; P, positive; N, negative; BT, before treatment; NA, nucleic acid; N/A, non-available.
