# Supplementary material for: Impaired Cellular Immunity to SARS-CoV-2 in Severe COVID-19 Patients
Source: Front Immunol. 2021 Feb 2;12:603563. doi: 10.3389/fimmu.2021.603563 (PMC7884325; doi:10.3389/fimmu.2021.603563)
Supplement: Supplementary file 3 [file Table_2.docx]

Table S2

**Table S2 Immune index for the severe COVID-19 patients**

| Pt# | NK% | NKT% | T% | CD4% | CD8% | CD4:CD8 | NAT50 |
| --- | --- | --- | --- | --- | --- | --- | --- |
| #1 | 1.31 | 0.96 | 80.3 | 70.2 | 21.8 | 3.22 | 23.04 |
| #2 | 1.38 | 0.28 | 69.2 | 83.7 | 11.6 | 7.22 | 475.5 |
| #3 | N/A | N/A | N/A | N/A | N/A | N/A | 1257 |
| #4 | N/A | N/A | N/A | N/A | N/A | N/A | 27.14 |
| #5 | N/A | N/A | N/A | N/A | N/A | N/A | 975.4 |
| #6 | 1.61 | 0.85 | 83.8 | 87.3 | 8.27 | 10.56 | 676.2 |
| #7 | 2.69 | 0.88 | 67.5 | 83.9 | 7.33 | 11.45 | 0 |
| #8 | 3.57 | 0.67 | 68 | 69 | 18.2 | 3.79 | 169.4 |
| #9 | 6.53 | 3.00 | 60.9 | 65.1 | 19.9 | 3.27 | 273.1 |
| #10 | 3.78 | 2.18 | 61.9 | 80.7 | 10.6 | 7.61 | 115 |

Notes: pt, patient; NAT50, neutralizing antibody titer; N/A, non-available
